# New Perspectives on Loneliness: From Micro Qualitative to Macro Quantitative Cross-National Comparisons

**DOI:** 10.1093/geroni/igaa057.2060

**Published:** 2020-12-16

**Authors:** Marja Aartsen

## Abstract

Longitudinal research revealed a number of micro-level drivers of loneliness, such as widowhood, exclusion from the wider society, ill health and migrant status, but a number of questions are still unanswered. For example, the prevalence of loneliness varies substantially across countries, but we do not know precisely what causes these differences. It may be due to differences in the composition of the populations, it may also be caused by macro-level drivers, or by variations in the impact of risk factors between countries. For example, losing a spouse may be loneliness provoking in countries where living with a partner is the norm, but less so in countries where living alone is more valued. Also how early childhood and events over the life course affect the level of loneliness in later life is still under-researched. The aim of our symposium is to address this gap by presenting different perspectives on loneliness and social isolation. The first presenter interprets five-year follow-up information from qualitative interviews with a life course perspective. The second investigates the role of trust as factor producing social integration, which leads to variations in loneliness. The third compares and discusses loneliness in three different continents, based on an ecological model of contexts. The forth presenter critically discusses ways to measure loneliness in societies that are culturally distinct from western cultures. The last presenter discusses the dynamics between loneliness and material deprivation in Europe. The findings provide a new lens through which we can understand loneliness and inform about effective prevention.

